# Corticosteroid-Induced Immunosuppression Ultimately Does Not Compromise the Efficacy of Antibiotherapy in Murine *Mycobacterium ulcerans* Infection

**DOI:** 10.1371/journal.pntd.0001925

**Published:** 2012-11-29

**Authors:** Teresa G. Martins, Gabriela Trigo, Alexandra G. Fraga, José B. Gama, Adhemar Longatto-Filho, Margarida Saraiva, Manuel T. Silva, António G. Castro, Jorge Pedrosa

**Affiliations:** 1 Life and Health Sciences Research Institute, School of Health Sciences, University of Minho, Braga, Portugal; 2 ICVS/3B's - PT Government Associate Laboratory, Braga/Guimarães, Portugal; 3 Institute for Biotechnology and Bioengineering, Centre of Biological Engineering, University of Minho, Braga, Portugal; 4 Laboratory of Medical Investigation 14, Faculty of Medicine of University of São Paulo, São Paulo, Brazil; 5 Molecular Oncology Research Center, Barretos, São Paulo, Brazil; 6 Institute for Molecular and Cell Biology, Porto, Portugal; Fondation Raoul Follereau, France

## Abstract

**Background:**

Buruli ulcer (BU) is a necrotizing disease of the skin, subcutaneous tissue and bone caused by *Mycobacterium ulcerans*. It has been suggested that the immune response developed during the recommended rifampicin/streptomycin (RS) antibiotherapy is protective, contributing to bacterial clearance. On the other hand, paradoxical reactions have been described during or after antibiotherapy, characterized by pathological inflammatory responses. This exacerbated inflammation could be circumvented by immunosuppressive drugs. Therefore, it is important to clarify if the immune system contributes to bacterial clearance during RS antibiotherapy and if immunosuppression hampers the efficacy of the antibiotic regimen.

**Methodology/Principal Findings:**

We used the *M. ulcerans* infection footpad mouse model. Corticosteroid-induced immunosuppression was achieved before experimental infection and maintained during combined RS antibiotherapy by the administration of dexamethasone (DEX). Time-lapsed analyses of macroscopic lesions, bacterial burdens, histology and immunohistochemistry were performed in *M. ulcerans*-infected footpads. We show here that corticosteroid-immunosuppressed mice are more susceptible to *M. ulcerans*, with higher bacterial burdens and earlier ulceration. Despite this, macroscopic lesions remised during combined antibiotic/DEX treatment and no viable bacteria were detected in the footpads after RS administration. This was observed despite a delayed kinetics in bacterial clearance, associated with a local reduction of T cell and neutrophil numbers, when compared with immunocompetent RS-treated mice. In addition, no relapse was observed following an additional 3 month period of DEX administration.

**Conclusions/Significance:**

These findings reveal a major role of the RS bactericidal activity for the resolution of *M. ulcerans* experimental infections even during immunosuppression, and support clinical investigation on the potential use of corticosteroids or other immunosuppressive/anti-inflammatory drugs for the management of BU patients undergoing paradoxical reactions.

## Author Summary

Buruli ulcer (BU) is an infectious disease caused by the environmental pathogen *Mycobacterium ulcerans* that affects the skin, subcutaneous tissue and bone, presenting extensive tissue necrosis. Standard treatment of BU patients consists of a combination of the antibiotics rifampicin and streptomycin (RS) for 8 weeks. Histological analysis of biopsies taken from the lesions of treated patients reveals an augmented inflammation that is suggested to contribute to the antibiotics' efficacy. However, in some patients, this inflammatory process developed during RS treatment may cause disease worsening, the so-called paradoxical reactions. By using a mouse model of *M. ulcerans* footpad infection, we show that mice co-administered with RS and the immunosuppressive/anti-inflammatory corticosteroid dexamethasone (DEX) are efficiently cured by the end of antibiotic treatment, although with a slight delay in bacterial clearance, pointing to a contribution of immune effector mechanisms. Additionally, no disease reactivation was observed after an additional period of 3 months of DEX administration. These findings have an important impact for the management of antibiotic-treated BU patients with paradoxical reactions, since the use of corticosteroids in mouse experimental infection do not cause treatment failure or disease reactivation, and therefore represents a potential strategy to control exacerbated immune responses during BU antibiotic treatment.

## Introduction

Buruli ulcer (BU) is a necrotizing disease of the skin, subcutaneous tissue and bone [1], [2]. The pathogenesis of the disease is associated with local and regional cytotoxic/immunosuppressive activities of the lipidic toxin mycolactone, produced by the environmental pathogen *Mycobacterium ulcerans* [3]–[7]. The clinical forms of BU disease are characterized by an initial nonulcerative lesion, often a nodule or a papule or the more disseminated forms plaques and oedema. Each of these forms can evolve to an ulcer and metastasize with the development of new cutaneous lesions or osteomyelitis [1], [2]. Established BU lesions are characterized by extensive necrotic, acellular areas with clumps of extracellular bacilli surrounded by a band of inflammatory cells, usually neutrophils and macrophages [8]–[10]. Although an extracellular localization of the bacilli is frequently seen in histological sections, *M. ulcerans* presents an intramacrophage growth phase in its life cycle before shedding to the extracellular compartment, and this supports the observation of intracellular bacilli at the peripheries of necrotic areas [11]. It has also been shown in the mouse model that, in addition to the site of infection, the draining lymph nodes (DLN) are colonized with bacilli, leading to extensive cell apoptosis, nodular tissue damage, and consequently depletion of *M. ulcerans*-specific T cells, further compromising the host immune response [12].

BU is a difficult-to-treat disease, however, improvement in case management has been achieved with the introduction of combined antibiotherapy with rifampicin and streptomycin (RS), a regimen recommended in 2004 by the World Health Organization (WHO) [13]. Successful results for the treatment of nonulcerative and small ulcers have been described [14]–[17], but variation in efficacy has been reported for advanced and disseminated lesions, for which surgery is still required in combination with antibiotherapy to achieve healing [15]–[19]. Subsequent to RS treatment, both in humans and in the mouse model, the immunosuppressive state at the *M. ulcerans* foci of infection wanes over time, a process characterized by an increase in inflammatory infiltrates, phagocytic activity and development of organized lymphoid structures [9], [20]–[22], which, in turn, is associated with a rapid decline of viable bacteria [20], [21]. Additionally, during antibiotherapy in experimental infections it has been shown that the structure of the DLN is preserved, contributing for the establishment of a cellular immune response at the site of infection [21]. Together, these observations implicate the host immune antimicrobial mechanisms in the process of mycobacterial killing during RS treatment.

Despite the efficacy of the RS antibiotic regimen, acid fast-bacilli (AFB) persist at the site of infection for extended periods of time [9], [14], [20]–[26]. Although these AFB are non-viable, as suggested by the non-reactivation of experimental infections after corticosteroid administration, mice maintain an inflammatory response with active phagocytes at the site of infection [21]. These observations in the mouse model, although not related with apparent pathology, are in line with the descriptions of paradoxical reactions occurring in some BU patients submitted to antibiotherapy. The so-called paradoxical reactions are characterized by exacerbated inflammatory responses and a surplus of degraded bacteria, which persist at the initial sites of treated lesions or in new cutaneous lesions [23], [26], [27]. These inflammatory responses are associated with a clinical worsening that follows an initial improvement of the lesion or even the appearance of fluctuant, erythematous and painful new lesions during or after antibiotic treatment [17], [23], [27], [28].

The occurrence of paradoxical reactions has also been described in *M. tuberculosis*-infected patients undergoing treatment [29]–[31]. In the case of *M. tuberculosis* infections, most presentations of paradoxical reactions are mild and do not require specific treatment or alteration in the antibiotic regimen [32], [33]. However, most severe cases, such as those that involve the central nervous system and pleural cavity, require treatment [33], [34]. Although the treatment of paradoxical reactions is not consensual [35], in part due to the lack of clinical trials, the use of corticosteroids seems to improve their resolution and the drug is usually used by clinicians [29], [33], . The use of corticosteroids has already been proposed for BU patients, in order to avoid or limit the extent of surgical management [27]. Corticosteroids are potent immunosuppressors and anti-inflammatory compounds, which act upon leukocyte circulation, function and migration to the sites of infection and tissue damage [36]–[38].

Considering the unknown contribution of the host effector immune mechanisms to the *M. ulcerans* killing observed during RS antibiotherapy, and its implications for the possible management of exacerbated inflammatory responses leading to paradoxical reactions through immunomodulation, we used the mouse model of *M. ulcerans* infection to address the impact of immunosuppression induced by dexamethasone (DEX) on the efficacy of RS treatment. For that, we evaluated the macroscopic progression of the lesions, bacterial burdens, histological alterations and occurrence of reactivation of infection after long-term DEX administration.

## Materials and Methods

### Ethics statement

This study was approved by the Portuguese national authority for animal experimentation Direcção Geral de Veterinária (ID: DGV 594 from 1^st^ June 2010). Animals were kept and handled in accordance with the guidelines for the care and handling of laboratory animals in the Directive 2010/63/EU of the European Parliament and of the Council.

### Animals

Eight-week-old female Balb/c mice were obtained from Charles River (Barcelona, Spain) and were housed under specific-pathogen-free conditions with food and water *ad libitum*.

### *M. ulcerans* experimental infection

*M. ulcerans* 98-912 (Institute of Tropical Medicine (ITM) collection, Antwerp, Belgium), a mycolactone D producing strain, was isolated in China from a case of ulcer and is highly virulent for mice, as previously described [6], [7], [8]. Preparation of the inoculum was performed as previously described [21]. Mice were inoculated in the left hind footpad with 0.03 ml of *M. ulcerans* suspension containing 5 log_10_ AFB, determined according to the method described by Shepard and McRae [39]. The right hind footpad was used as a control.

### Treatment of mice

Rifampicin and streptomycin (RS) were obtained from Sigma-Aldrich (USA). The dose and mode of administration were as previously described [21], [40]. Briefly, rifampicin was given orally by gavage at a dosage of 10 mg/kg of body weight and streptomycin was given by subcutaneous injection, at a dosage of 150 mg/kg of body weight. The treatment was initiated at the second week post-infection and was performed 6 days per week during 10 weeks. Antibiotic vehicles were given to control mice.

### Immunosuppressive treatment

**Figure 1:**
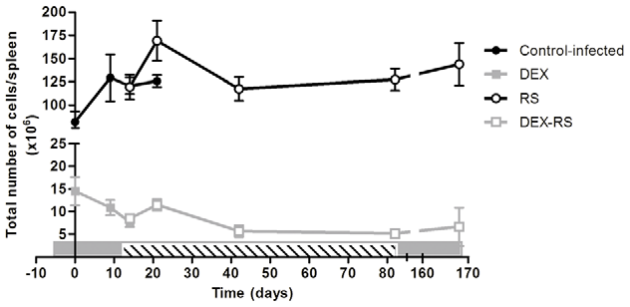
Total number of cells in the spleen of mice infected with *M. ulcerans*. Mice were administrated DEX (squares) or vehicle (circles) from day 6 before infection with *M. ulcerans* 98912 and were either left untreated (closed symbols) or treated with RS (open symbols) for 10 weeks. Grey bar represents the period of DEX administration. Striped bar represents the period of RS administration. Data points represent the mean ± SEM (n = 3–8).

Dexamethasone (DEX) (Sigma-Aldrich) was administrated by intraperitoneal injection at a dosage of 5 mg/kg of body weight, as previously described [21]. The administration was initiated at day 6 before *M. ulcerans* infection and lasted for 3 months after the end of antibiotic treatment, given 6 days per week. DEX vehicle was given to control antibiotic treated mice. Since DEX induces atrophy of the lymphoid organs (thymus, spleen and lymph nodes) in rodents [37], the kinetics of splenocytes was monitored as a readout of the immunosuppressive state. Approximately a ten to twenty-fold reduction in the total number of splenocytes was observed during the entire period of DEX administration to infected or infected and RS treated mice (Figure 1).

### Assessment of footpad swelling and bacterial growth

After infection, as an index of lesion development, footpad swelling of infected mice was determined over time, as previously described [8]. *M. ulcerans* growth in footpad tissues of infected mice was evaluated by colony forming units (CFU) at 9, 12, 14, 21, 42, 82 and 168 days post-infection. For the preparation of footpad suspensions, tissues were homogenized and decontaminated as previously described [8], [21], and serial dilutions were plated on 7H9 agar. CFU's were counted after 6–8 weeks of incubation at 32°C.

### Histological and immunohistochemical studies

Mouse footpads were harvested, fixed in buffered formalin and embedded in paraffin. Light-microscopy studies were performed on tissue sections stained with haematoxylin and eosin (HE) or Ziehl Neelsen (ZN), as previously described [8].

For immunohistochemistry, footpad tissue sections were deparaffinised and hydrated. Antigen retrieval was performed with EDTA 1 mM pH 8 or Borate buffer 0.02 M pH 7 for 30 min for the staining of T cells or neutrophils, respectively. Endogenous peroxidase activity was blocked with 0.3% hydrogen peroxide for 30 min and unspecific binding prevented by fetal bovine serum for 1 h, followed by 30 min blocking of avidin/biotin activity (Avidin/Biotin Blocking kit, Vector Laboratories, Inc.). Purified rat anti-CD3 (T cell marker, AbD Serotec) or purified rat anti-Ly-6G (neutrophil marker, BD Pharmingen) was added to the sections at a concentration of 1∶100 or 1∶1000, respectively, and incubated overnight at 4°C. Rabbit biotinylated anti-rat IgG antibody (Vector Laboratories, Inc.) was added at a concentration of 1∶200 for 1 h at room temperature, followed by 30 min of streptavidin-peroxidase polymer (Sigma-Aldrich). Staining was performed with DAB Peroxidase Substrate Kit, 3,3′-diaminobenzidine (Vector Laboratories, Inc.). Tissues were counter stained with haematoxylin and images were obtained with an Olympus BX61 microscope. The quantification of CD3^+^ T cells and Ly-6G^+^ neutrophils in the tissue sections was determined by counting the stained cells in the inflammatory area, using the software ImageJ. The values were represented as the mean cells per mm^2^ of inflammatory area of 5 images per section of total of 2 sections per footpad. Images were taken with a 20× objective lens.

### Determination of spleen cell counts

Single cell suspensions of the spleens from the different groups of mice were obtained and erythrocytes lysed with 0.87% ammonium chloride solution for 2 min at room temperature. Cells were counted using a haemocytometer.

### Statistical analysis

Differences between the means of experimental groups were analyzed with the two-tailed Student's *t* test, with a 95% level of significance, using the GraphPad Prism version 5.0 software. Differences with a *P* value<0.05 were considered significant.

## Results

### DEX-induced immunosuppression ultimately does not compromise *M. ulcerans* clearance during RS antibiotherapy

**Figure 2:**
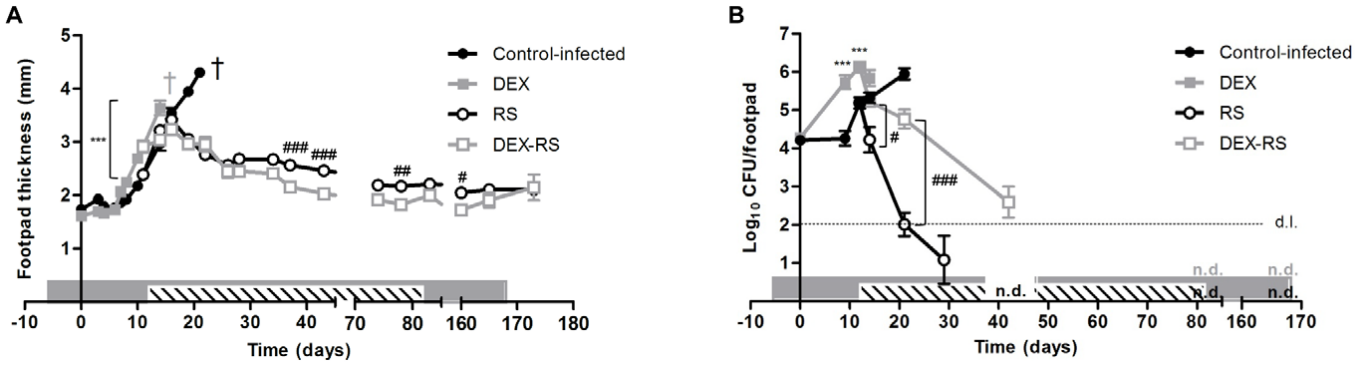
Lesion progression and bacterial proliferation in the footpad of mice infected with *M. ulcerans*. Mice were administrated DEX (squares) or vehicle (circles) and were left untreated (closed symbols) or treated with RS (open symbols) for 10 weeks. (A) Lesion progression was assessed by measurement of footpad swelling (n = 12–20). (B) Bacterial proliferation was assessed by CFU counts (n = 4–8). Asterisks represent significant differences between control-infected and DEX mice (***, *P*<0.001). Cardinals represent significant differences between RS and DEX-RS mice (^#^, *P*<0.05; ^##^, *P*<0.01, ^###^, *P*<0.001). Grey bar represents the period of DEX administration. Striped bar represents the period of RS administration. † Mice were euthanized for ethical reasons after the emergence of ulceration. n.d., not detected for the RS group of mice; n.d. (in grey), not detected for the DEX-RS group of mice. Data points represent the mean ± SEM.

To investigate the impact of corticosteroid-induced immunosuppression in the effectiveness of antibiotherapy against *M. ulcerans* infection, we used the experimental mouse model, treated or not with RS, in combination with DEX administration. As previously described [21], emergence of ulceration in the footpad of mice infected with virulent *M. ulcerans* 98-912 (control-infected mice) occurred at day 21 post-infection (Figure 2A), while RS administration in infected mice (RS mice), starting at day 12 post-infection, resulted in the continuing reduction of footpad swelling (Figure 2A) and viable bacteria in the subcutaneous tissue (Figure 2B), with complete clearance at the end of 10 weeks of treatment. To assess the protective role of host immunity in the early control of *M. ulcerans* proliferation, mice were administered with DEX from day 6 before infection until the end of the experimental period. Our results show that immunosuppressed mice (DEX mice) were more susceptible to infection, with faster progression of footpad swelling/ulceration (*P*<0.001 from day 8 to 14 post-infection) and higher bacterial loads (*P*<0.001) as compared to control-infected mice (Figure 2A and 2B).

To characterize the anti-*M. ulcerans* activity of the antibiotics in immunosuppressed hosts, DEX mice were subjected to the same antibiotic regimen as RS mice (DEX-RS mice). At the start of RS treatment, DEX mice presented a higher bacterial load as compared with control-infected mice (6.2 log_10_ CFU and 5.1 log_10_ CFU, respectively) (Figure 2B). During RS treatment, the progression of footpad swelling in the DEX-RS group followed the same trend as in RS mice, with a gradual decrease to basal levels, by the end of the RS administration period (Figure 2A). However, DEX-RS mice showed a delayed kinetics of bacterial clearance as compared to immunocompetent RS treated mice, with 2.6 log_10_ CFU at 42 days post-infection, time-point when CFU were already not detectable in the RS group (Figure 2B). Nevertheless, despite this delay, DEX-RS mice were able to clear the infection after a 10-week period of antibiotic regimen (Figure 2B). Moreover, the extension of DEX administration for 3 months after the completion of antibiotherapy did not result in disease reactivation (Figure 2A) nor in the detection of viable bacilli (Figure 2B), showing that the RS regimen is effective, even in corticosteroid-immunosuppressed hosts.

### DEX decreases the local inflammatory response to *M. ulcerans* infection developed during RS treatment

DEX-treated mice showed an increased susceptibility to infection by *M. ulcerans* strain 98-912 in terms of bacterial proliferation and emergence of ulceration. However, DEX-RS mice were able to clear bacteria, although with a delay, as compared to RS mice.

**Figure 3:**
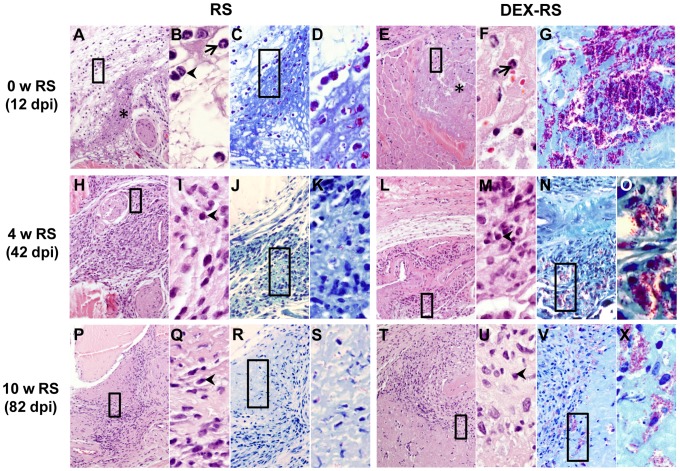
Histology of mice footpads infected with *M. ulcerans*. Histological sections of footpads collected at different time points were stained with HE (A, B, E, F, H, I, L, M, P, Q, T and U) or ZN (C, D, G, J, K, N, O, R, S, V and X). Magnifications: ×10 (A, E, H, L, P and T), ×20 (C, G, J, N, R and V), ×60 (B, D, F, I, K, M, O, Q, S, U and X). (A and E) Footpads of control-infected and DEX mice before the beginning of RS treatment (12 days post-infection), showing necrotic areas (asterisks). Magnifications of panel A and E (rectangles) show neutrophils (B and F; arrows) and mononuclear cells (B; arrowheads) adjacent/in necrotic areas. (C and G) Staining for bacteria in necrotic areas and (D) is a magnification of panel C. (H, L, P and T) Footpads of control-infected and DEX mice with 4 and 10 weeks of RS (42 and 82 days post-infection) show abundant cellular infiltration, composed mainly by mononuclear cells (I, M, Q and U; arrowheads). (J, N, R and V) Staining for bacteria in the same tissue areas and magnifications of the bacilli are shown in panels K, O, S and X. w, weeks; dpi, days post-infection.

To assess the contribution of immune mechanisms to the clearance of *M. ulcerans*, we analyzed the histopathology at the site of infection in immunocompetent vs. DEX-treated mice. As previously described [21], at day 12 post-infection the presence of central necrotic areas with extracellular bacilli surrounded by a predominantly neutrophilic/macrophagic infiltrate (Figure 3A–D) are histological features of a progressive subcutaneous infection with virulent *M. ulcerans* 98-912. On the other hand, during RS treatment we observed a switch of the inflammatory profile to abundant lymphocytic/macrophagic infiltrates, which was maintained until the end of the experimental period (10 weeks post-infection) (Figure 3H–I and 3P–Q).

In comparison to control-infected mice (Figure 3A), footpad tissue of DEX mice presented widespread necrosis (Figure 3E) associated with massive clumps of extracellular bacilli (Figure 3G), which is consistent with the higher bacterial burden (Figure 2B). The pattern of the inflammatory response in this group of immunosuppressed mice was similar to control-infected mice, with neutrophils adjacent and/or in necrotic areas (Figure 3B and 3F).

In immunosuppressed mice submitted to antibiotherapy (DEX-RS mice) after 4 weeks of RS administration (42 days post-infection), the increased bacterial burdens, as compared with RS mice, was reflected in the higher number of clumps of extracellular bacilli (Figure 3J–K and 3N–O). Despite the higher bacterial burden, inflammatory infiltrates showed a similar profile to immunocompetent RS mice (Figure 3H–I and 3L–M), characterized by an increase of a predominantly mononuclear infiltrate, as compared to non-treated mice (Figure 3E–F). This profile was maintained at the end of treatment (Figure 3P–Q and 3T–U).

**Figure 4:**
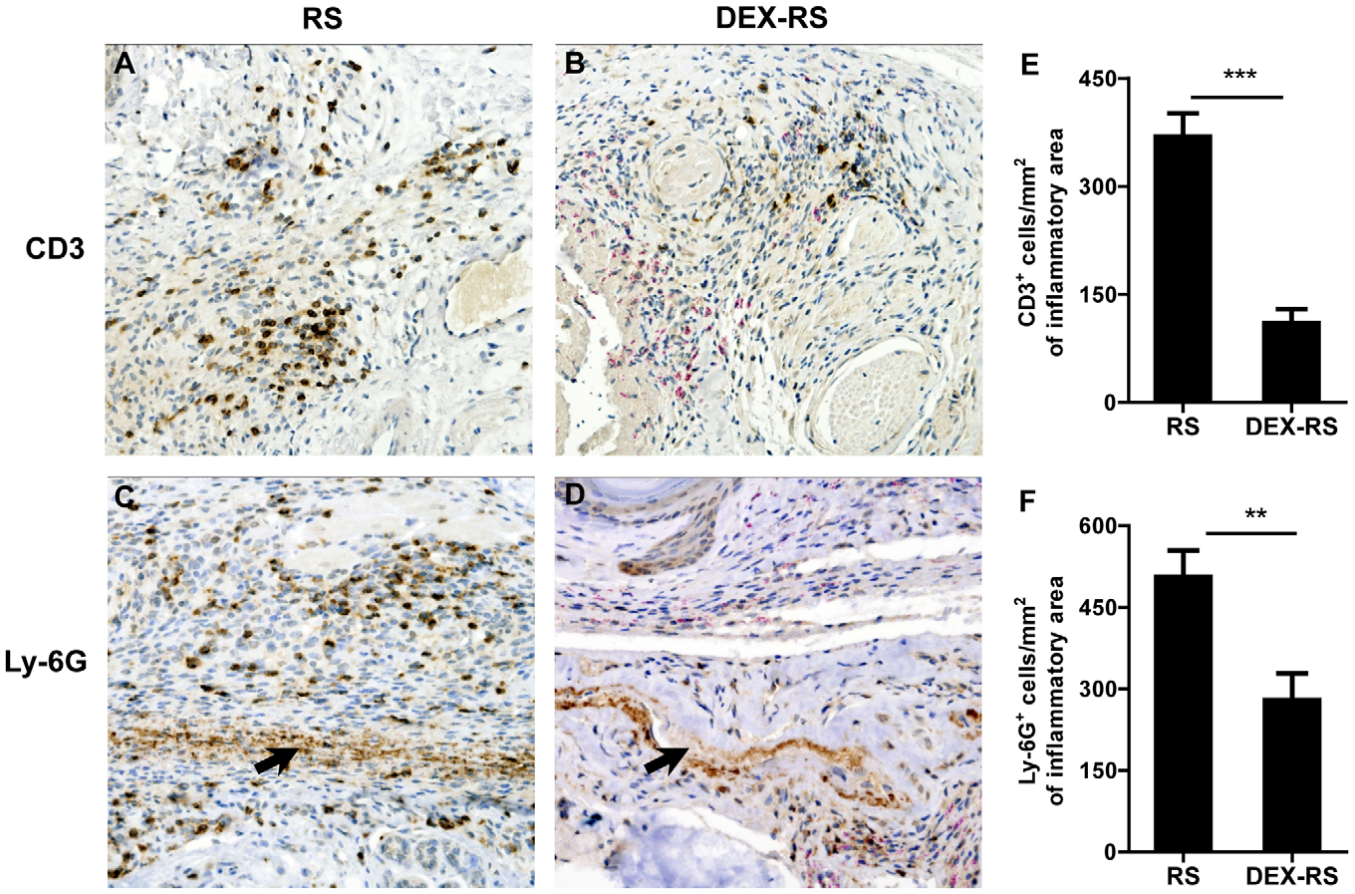
Immunohistochemistry of mice footpads infected with *M. ulcerans*. Histological sections of footpads of control-infected and DEX mice at 4 weeks of RS treatment (42 days post-infection) were stained for the antigen marker CD3 of T cells (A and B) or Ly-6G of neutrophils (C and D), and with ZN to visualize the bacilli. Magnifications: ×20. Footpads of RS mice (A) show increased staining for T cells in comparison to DEX-RS mice (B). Staining for Ly-6G in RS mice (C) is located in necrotic areas (arrow) and in cells interspersed in the inflammatory infiltrates. In comparison, footpads of DEX-RS mice (D) show staining for Ly-6G mainly in necrotic areas (arrow), and fewer stained cells appear scattered in the tissue. The number of CD3 (E) and Ly-6G positive cells (F) per mm^2^ of inflammatory area in the stained tissue sections was quantified by using a 20× objective lens. Asterisks represent significant differences between RS and DEX-RS mice (**, *P*<0.01; ***, *P*<0.001). Data points represent the mean ± SEM of 2 different histological sections of each mouse footpad sample, in a total of 3 footpads per group.

Given the known immunosuppressive and anti-inflammatory properties of DEX, namely the inhibition of inflammatory cell recruitment, including neutrophils and lymphocytes, to the focus of infection [37], [38], [41], [42], we next analyzed if there were differences in these cell populations in infected footpads. We observed that after 4 weeks of RS treatment (42 days post-infection), despite the similar amounts of inflammatory infiltrates observed in slides stained with HE (Figures 3H–I and 3L–M), DEX-RS mice presented a lower number of T cells stained by immunohistochemistry (Figure 4B) as compared with RS mice (Figure 4A). The quantification of T cells confirmed the histological observations, with a median distribution of 369 cells/mm^2^ of inflammatory area in RS mice, whereas the DEX-RS group only showed 110 cells/mm^2^ (Figure 4E; *P*<0.001) In addition, in the DEX-RS group, most of the staining for the neutrophilic marker Ly-6G was observed in the remaining necrotic tissue with cell debris, and only few intact cells were stained in the peripheries of the lesion (Figure 4D), when compared with RS mice for which intact neutrophils were mainly found at the peripheral areas (Figure 4C). The quantification of these cells showed a distribution of 506 vs. 279 cells/mm^2^ of inflammatory area in the RS and DEX-RS group of mice, respectively (Figure 4F; *P*<0.01).

These data show that corticosteroid-induced immunosuppression, associated with increased *M. ulcerans* proliferation, results in increased necrosis at infection foci. Following RS administration, bacterial clearance ensues in immunosuppressed mice, although with slower kinetics, which is associated with lower T cell numbers.

## Discussion

The recent regimen with RS, introduced by the WHO in 2004, has been proven effective in BU patients with nonulcerative or small ulcers, but variation in efficacy is reported for more advanced lesions [14]–[19]. Improvements to this protocol have been tested, such as the introduction of a fully oral antibiotic regimen replacing streptomycin by clarithromycin, since streptomycin cannot be administered to pregnant women or intolerant patients, and demands daily intramuscular administration [16],[26],[43]–[46]. Regardless the antibiotic protocol, there have been reported cases of clinical worsening of the lesions after an initial period of improvement. These so-called paradoxical reactions have been attributed to an exacerbated inflammatory response to mycobacterial antigens resulting from effective antibiotic activity [17], [23],[26]–28. In fact, several studies in humans and in mice have shown an increase of the local immune response during the antibiotic treatment, with abundant lymphocyte/macrophage infiltrates, in some cases forming organized lymphoid structures, and with the phagocytosis of bacilli [9], [20]–[22]. In addition, dead bacilli persist at the site of the treated lesion, which allows the maintenance of the inflammatory response [21]. Therefore, it is important to understand how far the immune response can be modulated, in order to regulate the paradoxical reactions without compromising the efficacy of the antibiotic treatment [9], [21], [22]. To address this question, we used a mouse model of *M. ulcerans* infection to characterize the contribution of the host immune response to the RS-associated clearance of *M. ulcerans*, as well as to study the impact of immunosuppression in the efficacy of the RS regimen. For that, mice were systemically immunosuppressed with the synthetic corticosteroid DEX and treated with RS during a period of 10 weeks. We observed that, even in a state of induced immunosuppression, RS-treated mice are able to clear the infection, although with a delayed kinetics, with no relapse following more 3 months of DEX administration.

DEX is one of the most powerful corticosteroid immunosuppressant drugs, with activity on leukocyte circulation, function and migration to the sites of infection [36]–[38]. In our model, this drug proved to be immunosuppressive, since its continuous administration, initiated 6 days before the inoculation of *M. ulcerans*, rendered mice more susceptible to infection by the virulent isolate 98-912, with a faster progression of macroscopic pathology and increased bacterial burdens. Accordingly, we observed a high reduction in the total number of splenocytes in DEX and DEX-RS mice during the entire period of DEX administration. Moreover, the administration of DEX also induced a reduction of local T cells (CD3 positive) during antibiotherapy. It is known that *M. ulcerans* infection induces the activation of IFNγ-specific T cells that are later depleted, locally and regionally, due to the cytotoxic activity of mycolactone [12]. This Th1 type of immune response was proven to be important for protection against *M. ulcerans* strains of lower virulence, as shown by the higher susceptibility of mice deficient in either T cells or IFNγ [7], [12]. Therefore, the lower number of T cells in the footpads of mice treated with DEX is expected to contribute to the host susceptibility to infection in the present model. On the other hand, T cell survival is allowed during RS administration, in association with the decline of viable bacilli.

The fact that DEX-RS mice presented a delayed clearance of viable bacteria in the footpad lesions suggests a role of the immune response in the efficacy of the antibiotic regimen. Such a type of immune participation is suggested in another experimental model of antibiotherapy in mice infected with *Mycobacterium avium* complex, where treatment with sparfloxacin and ethambutol is enhanced by combination with an inhibitor of the cortisol receptor [47]. However, it is also important to stress that, at the beginning of the RS regimen, immunosuppressed mice already presented a higher bacterial burden associated with more severe histopathology, which may also hamper the diffusion of the antibiotics to the core of the lesion. Nevertheless, despite the higher bacterial load and suppressed local inflammatory responses in the footpad of immunosuppressed mice (with lower numbers of T cells and neutrophils), the antibiotic regimen was able to clear the infection after 10 weeks of administration. Indeed, no relapse was observed after an additional 3 months of DEX administration. This points out that the bactericidal activity of the drug is the main factor in the resolution of the infection.

In addition to the analysis of the treatment efficacy in mice administered with DEX, it would have been interesting to test specifically if corticosteroids or other immunosuppressive/anti-inflammatory drugs could control paradoxical reactions during or after antibiotherapy. However, there is currently no proper animal model to study paradoxical reactions. Corticosteroids are being successfully used in the management of other types of paradoxical reactions, for instance in patients with tuberculosis presenting severe forms that must be treated, and when surgery of the affected area is unwanted or difficult/risky to perform, such as in the central nervous system [29], [34]. The fact that the antibiotic treatment was efficient in our model, even in mice administered with DEX, suggests that the use of corticosteroids in BU patients undergoing severe paradoxical reactions may not represent a risk of reactivation/treatment failure. However, more studies are needed to address this point, especially when regarding the management of more severe lesions, where culture positivity is sometimes detected at the end of antibiotic treatment [16], [17]. On the other hand, monitoring the persistence of AFB in lesions is also a feature to be considered for the management of BU patients with paradoxical reactions submitted to corticotherapy, since the end of the immunosuppressed-induced state could be followed by an exacerbated up-regulation of the immune response.

Although the use of corticosteroids in our mouse model does not compromise the efficacy of antibiotic treatment, we should stress that in humans the use of these drugs should be considered with caution. Several side effects are associated with corticosteroids, such as the development of metabolic alterations like hyperglycemia or adrenal atrophy, or even impaired wound healing, but these effects are dependent on the type of corticosteroid used, doses and the time of administration [48], [49]. Patients receiving corticosteroids are also at risk of developing opportunistic or reactivating infections, like strongyloidiasis, tuberculosis, fungal infections and cytomegalovirus [48], [50]. However, a randomized placebo-controlled clinical trial in South Africa on the systemic use of corticosteroids to control paradoxical tuberculosis-associated immune-reconstitution inflammatory syndrome in HIV-infected patients receiving antitubercular and antiretroviral therapy, showed beneficial activity in ameliorating the symptoms with minimal side-effects, when a low and short-term therapy with prednisone was used [51]. The authors advise, though, that excluded diagnosis of multidrug-resistant tuberculosis or Kaposi's sarcoma should be performed before starting corticosteroids [51]. Therefore, a possible use of corticosteroids in BU patients or other alternative management strategies justifies clinical investigation and deserves consideration, depending on the severity of the case, potential side effects and evaluation of the risk/benefit ratio. Like in tuberculosis patients, paradoxical reactions in BU patients are transient, but in some cases these result in a considerable enlargement of the lesions and a prolonged period to achieve healing [28]. A strategy to avoid or improve such outcome during or after antibiotherapy would be desirable. Although we did not test other immunosuppressive/anti-inflammatory drugs, our study may also open possibilities to study the management of more severe paradoxical reactions with drugs that, for instance, could be applied locally, thus minimizing systemic effects and avoiding the need of surgery.

In summary, corticosteroid-induced immunosuppression during experimental *M. ulcerans* infection, although delaying bacterial clearance, does not ultimately compromise the efficacy of the WHO recommended RS regimen. This observation may be explained by a major role of the bactericidal activity of RS that overlaps the activity of the local immune response. This study justifies future clinical studies on the potential use of corticosteroids or other immunosuppressive/anti-inflammatory drugs in the management of BU patients undergoing paradoxical reactions.
